# Potential multifunctional agents with anti-hepatoma and anti-inflammation properties by inhibiting NF-кB activation

**DOI:** 10.1080/14756366.2019.1635124

**Published:** 2019-07-10

**Authors:** Chang-Ming Su, Gui-Ge Hou, Chun-Hua Wang, Hong-Qin Zhang, Cheng Yang, Mei Liu, Yun Hou

**Affiliations:** a School of Pharmacy, The Key Laboratory of Prescription Effect and Clinical Evaluation of State Administration of Traditional Chinese Medicine of China, Binzhou Medical University, Yantai, PR China;; b School of Basic Medical Sciences, Binzhou Medical University, Yantai, PR China;; c Pharmacy Department, The Second People’s Hospital of Dongying, Dongying, PR China

**Keywords:** 3,5-Bis(arylidene)-4-piperidones, anti-cancer, anti-inflammation, NF-κB inhibitor, molecular docking

## Abstract

Inhibition of NF-κB signalling has been demonstrated as a therapeutic option in treating inflammatory diseases and cancers. Herein, we synthesized novel dissymmetric 3,5-bis(arylidene)-4-piperidones (BAPs, **83–102**) and characterized fully. MTT and ELISA assay were performed to screen the anti-hepatoma and anti-inflammation properties. **96** showed the most potential bioactivity. **96** could promote HepG2 apoptosis through up-regulating the expression of C-Caspase-3 and Bax, down-regulating the expression of Bcl-2, while markedly inhibit LPS or TNF-*α*-induced activation of NF-*κ*B through both inhibiting the phosphorylation of IκBα and p65, and preventing the p65 nuclear translocation to exhibit both anti-hepatoma and anti-inflammatory activities. Molecular docking verified that simulated **96** can effectively bond to the active site of Bcl-2 and NF-κB/p65 proteins. **96** inhibited xenografts growth by reducing the expression of TNF-α and Bcl-2 in the tumour tissue. This study suggested that **96** could be developed as a potential multifunctional agent for treatment of inflammatory diseases and cancers.

## Introduction

**Figure 1.**
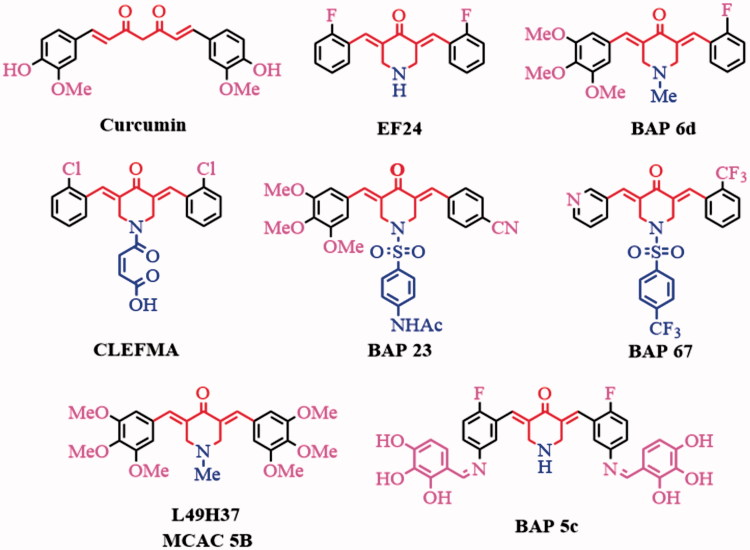
The structures of curcumin and some reported BAPs.

Curcumin (Figure 1), a major active ingredient of turmeric, has been demonstrated showing anti-inflammatory and anti-cancer activities^1^. However, due to its poor solubility, unstability and low bioavailability, its clinical utility is limited^2^^,^^3^. Therefore, a lot of its analogues were developed. 3,5-bis(arylidene)-4-piperidone derivatives (BAPs) possess the 1,5-diaryl-3-oxo-1,4-pentadienyl pharmacophore leads a priorchemosensitivity to cancer cells rather than normal cells^4^^,^^5^. 3,5-Bis(2-flurobenzylidene)piperidin-4-one (EF24, Figure 1) inhibits cancer growth and metastasis through inhibiting NF-*κ*B pathways^6^^,^^7^. CLEFMA induces cell apoptosis of lung cancer xenografts by inhibiting Bcl-2 expression and up-regulating Bax expression^8^. L49H37 (or MCAC 5B, Figure 1) exhibits better inhibitory effects on cell cycle of pancreatic stellate cells (PSCs), as well as anti-lung cancer activity, and chemosensitization based on ROS-mediated JNK pathway activation and NF-*к*B pathway inhibition^9^**^–^**^11^.

In recent years, we have focused on improving modifying the structure of BAPs to optimize the anti-inflammation and anti-hepatoma capability by inhibiting the NF-*κ*B signalling. We have reported some symmetric or dissymmetric *N*-substituted-BAPs^12^**^–^**^17^and Schiff-base substituted BAPs^18–21^ with improved anti-hepatoma and anti-inflammation activities. For example, the dissymmetric BAP (*3E,5E*)-3-(2-fluorobenzylidene)-5-(3,4,5-trimethoxybenzylidene)-1-methylpiperidin-4-one(BAP **6d**, Figure 1) with different substituent groups on both sides can potentially inhibit HepG2 and THP-1 growth, which were proved by inducing cell apoptosis using flow cytometry and suppressing the growth of HepG2 xenografts^12^. The further optimized and synthesized dissymmetric *N*-phenylsulphonyl-BAPs **23** and **76** can prevent the nuclear translocation of NF-*κ*B induced by inflammatory cytokines (lipopolysaccharide (LPS), TNF-*α*) and exhibit both anti-inflammation and anti-cancer capability in hepatic carcinoma cell lines^13^^,^^14^. However, the bioactivity is still not satisfied and the toxicity to the normal cell may be optimized.

Schiff-base compounds with active –C=N groups exhibit a range of biological activities, including antibacterial, antiviral, anti-cancer and anti-inflammatory properties^22^^,^^23^. We tried to incorporate of amino-substituted BAPs and aromatic aldehydes substituted by -X or -OH to construct novel symmetric^18^ (such as BAP **5c**) or dissymmetric Schiff-base substituted BAPs with desired anti-cancer and anti-inflammation activity. In this study, a series of novel dissymmetric BAPs **83–102** were synthesized, characterized and evaluated as potential anti-hepatoma and anti-inflammatory agents.

## Experimental

### Materials and methods

*N*-Methyl-4-piperidinone, stannous chloride and allaromatic aldehydes were purchased from Sinopharm Chemical Reagent Co. Ltd (Shanghai, China) and used as obtained without further purification. Compounds (3*E*,5*E*)-3–(2-fluorobenzylidene)-1-methyl-5–(3-nitrobenzylidene)piperidin-4-one (**83**), (3*E*,5*E*)-3-(3-aminobenzylidene)-5-(2-fluorobenzylidene)-1-methylpiperidin-4-one (**92**) were prepared based on a literature^19^. NMR data were collected using a Bruker Avance 400 MHz for ^1^H NMR with chemical shifts *δ* relative to TMS, while ^13^C NMR data were collected at 100 MHz on a Bruker Avance 400 MHz spectrometer or 150 MHz on a Bruker Avance 600 MHz spectrometer (Bruker Scientific, Billerica, MA). The HREIMS data were obtained on a Finnigan-MAT-95 mass spectrometer (Bruker Scientific, Billerica, MA).

### Preparation of 84-91

*N*-Methyl-4-piperidinone (1.13 g, 10.0 mmol) and two kinds of aromatic aldehydes (10.0 mmol, respectively) mixed with 20 ml glacial acetic acid. Dry hydrogen chloride gas was bubbled into this mixture for about 45 min (min), and the mixture was continuously stirred at room temperature (RT) for 24 h (monitored by TLC). The precipitate was collected, and then dissolved in distilled water (50 ml). Aqueous NaOH solution was added until the pH of the system was adjusted to 7. The system was filtered and subsequently washed by distilled water to provide a yellow precipitate. The precipitate was purified on silica gel by column using methanol/petroleum ether/EtOAc (10:10:1, v/v/v) as the eluent to afford yellow powders **84–91**.

### Preparation of 93-102

BAP **92** (0.32 g, 1.0 mmol) and aromatic aldehydes (1.0 mmol) were dissolved in methanol (5 ml). A drop of formic acid was added into the mixture. The mixture was stirred for 3 h at ambient temperature (monitored by TLC). The precipitate was collected and recrystallized from methanol to afford yellow crystals of **93–102**.

### Anticancer testing with MTT method

Compounds **83–102** were screened against human neoplastic cell lines, such as human breast cancer cells (MCF-7), human colon cancer cells (HCT116), human lung carcinoma cells (A549), human gastric adenocarcinoma cells (SGC7901), human liver hepatocellular carcinoma cell line (HepG2), human cervical carcinoma cells (HeLa), human acute mononuclear granulocyte leukaemia (THP-1), and human normal hepatic cell line (LO2) using modified MTT assay^18^. All the cells were obtained from the American Type Culture Collection (ATCC, Manassas, VA). The cells were cultured in DMEM or 1640. Positive controls (Doxorubicin (DOX) and Curcumin) and BAPs were initially dissolved in dimethyl sulphoxide (DMSO). Experimental concentration of DMSO was always 0.1% (v/v). The concentrations of BAPs and positive controls were 50, 20, 10, 5, 2.5, 1, 0.5, 0.1, 0.05, and 0.01 μg/mL.

The cells (1 × 10^4^ cells/well) were seeded in a 96-well plate and incubated for 24 h. Cells treated with serial concentrations of drugs were incubated for another 24 h. After the media removed, 20 μL of MTT (5 mg/mL) solution was added and incubated for 4 h at 37 °C. The MTT solution was removed and then 150 μL of DMSO was added. The optical density was measured with a multi-well plate reader (TECAN, Mӓnnedorf, Switzerland) at 570 nm. The results are expressed as a decrease in the cell viability (%) in comparison to untreated controls. The concentration of each compound was examined in triplicate, and the IC_50_ values are expressed in Table 1.

**Table 1.** Cytotoxicity of BAPs, Curcumin, and DOX.

Compounds	R_1_	R_2_ or R_3_	MCF-7		HCT116		A549		SGC7901		HepG2		HeLa		THP-1		LO2
			IC_50_ (μM)	SI^a^	IC_50_ (μM)	SI^a^	IC_50_ (μM)	SI^a^	IC_50_ (μM)	SI^a^	IC_50_ (μM)	SI^a^	IC_50_ (μM)	SI^a^	IC_50_ (μM)	SI^a^	IC_50_ (μM)
**83**	2-F	3-NO_2_	7.2 ± 0.3	1.8	6.2 ± 0.6	2.1	2.3 ± 0.2	5.7	3.7 ± 0.3	3.5	1.2 ± 0.2	10.9	2.5 ± 0.3	5.2	1.3 ± 0.1	10.1	13.1 ± 0.8
**84**	2,4-F	3-NO_2_	8.5 ± 0.2	2.3	9.5 ± 0.1	2.0	6.2 ± 0.3	3.1	7.6 ± 0.8	2.5	8.2 ± 0.3	2.4	10.2 ± 0.2	1.9	11.8 ± 0.2	1.6	19.3 ± 0.3
**85**	3,5-F	3-NO_2_	18.5 ± 0.4	1.1	13.2 ± 0.5	1.5	9.7 ± 0.2	2.1	24.5 ± 0.8	0.8	14.3 ± 0.1	1.4	14.2 ± 0.6	1.4	5.4 ± 0.3	3.8	20.3 ± 0.1
**86**	2,5-F	3-NO_2_	11.1 ± 0.7	1.9	10.5 ± 0.9	2.0	9.3 ± 0.4	2.3	20.5 ± 0.5	1.0	9.2 ± 0.4	2.3	11.5 ± 0.8	1.9	7.0 ± 0.2	3.0	21.3 ± 0.4
**87**	2-Br-4-F	3-NO_2_	11.6 ± 0.3	1.2	7.7 ± 0.4	1.8	4.3 ± 0.3	3.2	4.7 ± 0.3	2.9	5.4 ± 0.5	2.5	9.4 ± 0.5	1.5	6.6 ± 0.1	2.1	13.7 ± 0.5
**88**	2-Br-4-F	3,4,5-OMe	6.3 ± 0.4	2.3	6.5 ± 0.3	2.3	5.6 ± 0.3	2.6	3.6 ± 0.1	4.1	5.3 ± 0.2	2.8	5.3 ± 0.3	2.8	7.6 ± 0.2	1.9	14.8 ± 0.3
**89**	2-OMe	3,4,5-OMe	9.7 ± 0.4	1.4	5.6 ± 0.1	2.4	4.9 ± 0.1	2.8	3.3 ± 0.3	4.1	4.3 ± 0.1	3.2	5.3 ± 0.4	2.6	7.9 ± 0.3	1.7	13.6 ± 0.1
**90**	4-OMe	3,4,5-OMe	15.5 ± 0.9	1.1	12.1 ± 0.4	1.4	5.8 ± 0.2	2.8	7.0 ± 0.4	2.4	8.2 ± 0.6	2.0	15.5 ± 0.2	1.1	5.0 ± 0.3	3.3	16.5 ± 0.1
**91**	4-OMe	3,4-Cl	12.5 ± 0.5	1.1	18.1 ± 0.2	0.8	9.2 ± 0.1	1.5	6.4 ± 0.1	2.2	4.5 ± 0.7	3.2	17.0 ± 1.3	0.8	7.2 ± 0.2	2.0	14.2 ± 0.3
**92**	2-F	3-NH_2_	2.8 ± 0.3	6.8	3.1 ± 0.3	6.1	2.2 ± 0.1	8.6	2.8 ± 0.2	6.8	1.1 ± 0.1	17.2	2.5 ± 0.2	6.1	1.2 ± 0.2	15.8	18.9 ± 0.7
**93**	2-F	2-OH-5-F	3.8 ± 0.9	4.0	2.5 ± 1.8	6.0	3.7 ± 0.1	4.1	2.8 ± 0.5	5.4	2.2 ± 0.2	6.9	3.0 ± 0.1	5.0	2.4 ± 0.5	6.3	15.1 ± 0.7
**94**	2-F	4-Cl	6.5 ± 0.1	2.8	6.3 ± 0.1	2.9	5.9 ± 0.3	3.1	6.4 ± 0.1	2.8	4.8 ± 0.1	3.8	11.1 ± 0.3	1.6	4.0 ± 0.1	4.5	18.0 ± 0.3
**95**	2-F	3,4-Cl	5.4 ± 0.2	2.0	6.3 ± 0.2	1.7	3.1 ± 0.2	3.5	3.3 ± 0.5	3.3	2.5 ± 0.1	4.4	4.3 ± 0.1	2.6	3.8 ± 0.1	2.9	11.0 ± 0.4
**96**	2-F	2-OH-5-Br	2.5 ± 0.2	8.0	2.5 ± 0.1	8.0	1.6 ± 0.4	12.4	2.4 ± 0.8	8.3	1.0 ± 0.4	19.9	2.1 ± 0.8	9.5	1.1 ± 0.6	18.1	19.9 ± 0.4
**97**	2-F	2-F-5-NO_2_	10.7 ± 0.1	1.1	11.7 ± 0.2	1.0	5.7 ± 0.4	2.1	3.4 ± 0.8	3.5	4.2 ± 0.5	2.8	5.1 ± 1.2	2.3	8.1 ± 0.4	1.5	11.8 ± 0.4
**98**	2-F	2,4-OH	10.3 ± 0.7	1.7	11.4 ± 0.7	1.5	3.2 ± 0.7	5.5	10.9 ± 0.3	1.6	7.5 ± 0.1	2.3	6.2 ± 0.1	2.8	5.1 ± 0.2	3.5	17.6 ± 0.2
**99**	2-F	2,5-OH	5.9 ± 0.2	2.6	7.42 ± 0.1	2.1	4.4 ± 0.1	3.5	4.7 ± 0.3	3.3	8.2 ± 0.1	1.9	15.3 ± 1.1	1.0	7.8 ± 0.4	2.0	15.6 ± 0.4
**100**	2-F	2,3,4-OH	7.7 ± 0.2	2.6	11.9 ± 0.1	1.7	1.9 ± 0.2	10.4	3.6 ± 0.1	5.5	8.8 ± 0.4	2.3	11.9 ± 0.2	1.7	7.7 ± 0.1	2.6	19.8 ± 0.3
**101**	2-F	2-OH-4-OMe	4.7 ± 0.2	3.0	4.9 ± 0.1	2.8	3.5 ± 0.2	4.0	10.8 ± 0.1	1.3	4.8 ± 0.1	2.9	8.9 ± 0.2	1.6	3.5 ± 0.2	4.0	13.9 ± 0.3
**102**	2-F	2-OH-3-Br-5-OMe	15.5 ± 1.2	1.0	18.1 ± 1.9	0.8	13.4 ± 0.7	1.1	7.0 ± 0.4	2.1	5.9 ± 0.1	2.5	9.1 ± 0.2	1.6	5.0 ± 0.2	3.0	14.8 ± 0.1
DOX	–	–	2.8 ± 0.7	1.7	3.5 ± 0.1	1.3	3.4 ± 0.4	1.4	3.3 ± 0.5	1.4	1.2 ± 0.1	3.9	7.0 ± 0.3	0.7	9.5 ± 0.8	0.5	4.7 ± 0.7
Curcumin	–	–	20.5 ± 0.2	1.4	17.2 ± 0.3	1.6	22.7 ± 1.0	1.2	22.8 ± 0.6	1.2	20.7 ± 0.1	1.4	19.4 ± 0.6	1.5	20.8 ± 0.6	1.4	28.3 ± 1.2

a The letters SI refer to the selectivity index which is the quotient of the IC_50_ values towards non-malignant LO2 cells.

### Anti-inflammation testing of BAPs

The anti-inflammatory activities of BAPs were evaluated by ELISA. After pretreated with 30 μM of positive drug pyrrolidinedithiocarbamate (PDTC) or 6 μM of BAPs for 2 h, cells were treated with LPS (1.0 μg/mL) for 22 h. The culture media were collected and centrifuged for detecting the expression levels of TNF-α by an ELISA kit (eBioScience, San Diego, CA).

### Western blot assay

The HepG2 cells were treated with BAP **96** at different concentrations (1.0, 2.0, and 4.0 µM) for 3 h, and washed twice by PBS. The cells were lysed and the extracted proteins of cell lysates were separated by 10% SDS-PAGE gel electrophoresis, then transferred onto polyvinylidene fluorides membranes. The membranes were probed with antibodies and visualized using an enhanced chemiluminescence (ECL) detection kit.

### Molecular docking study

The study was performed under CDOCKER and LibDock protocol of Discovery Studio 2017 R2. The 3D structure of all BAPs was generated and minimized. The crystal structures of Bcl-2 (PDB1YSW) and NF-κB/p65 (PDB 1MY5) were obtained from Protein Data Bank. They were optimized by hydrogenation, dehydration and CHARMm force field. The ligand of the Bcl-2 protein was removed, then the BAPs were place into the site sphere. Similarly, NF-κB/p65 protein was defined as receptor, and the site sphere was selected on the p65 NLS polypeptide, and then BAP **96** was placed. Molecular docking scoring is obtained by CDOCKER and LibDock program, respectively. The types of interactions between proteins and BAPs were analyzed at the end of the molecular docking.

### Apoptosis assay using annexin V-FITC staining

After treated with BAP **96** (1.0, 2.0, and 4.0 μM) and DMSO for 24 h. HepG2 cells were washed and added with 500 μL of Binding Buffer, 5 *μ*L of Annexin V-EGFP and 5 *μ*L of Propidiumlodide and mix (BD Biosciences, San Jose, CA) according to the manufacturer’s instruction. The mixed solution was gently vortexed and incubated in the dark at RT for 15 min. The results were detected by flow cytometry (BD FACS Calibur, San Jose, CA) within 1 h.

### Immunofluorescence staining

RAW264.7 cells were pretreated in 96-well plates with LPS (1 μg/mL) for 15 min and then treated with **96** for 2 h. Similarly, HepG2 cells were pretreated with TNF-α (10 ng/mL) for 15 min and then treated with **96** for 4 h. The cells were fixed with 2% paraformaldehyde for 15 min at 37 °C and permeabilized using 0.1% Triton X-100 for 10 min at RT. After blockage with 3% BSA for 30 min, the cells were incubated with anti-NF-κB p65 antibody (NOVUS BIOLOGICALS, Colorado, USA) at 4 °C overnight. After washing, the anti-mouse Alexa488-conjugated secondary antibody (Jackson Immuno Research, West Grove, PA) was incubated for 30 min at RT. After washed with PBS, the cells were incubated in 4-6-diamidino-2-phenyindole (DAPI; Sigma, St. Louis, MO) for 15 min in the dark at RT. The cell images were simultaneously viewed using a microscopy system in High Throughput Screen (Operetta, PerkinElmer, Waltham, MA).

### *In vivo* anti-hepatoma efficacy

The HepG2 cells xenografts were established using the Balb/c nude mice (female, 7-week-old, 17–19 g, *n* = 30) (Shanghai Laboratory Animal Center, Shanghai Shi, China) were used to investigate the anti-hepatoma effect of **96**. Animal experiments were reviewed and approved by the Binzhou Medical University Experimental Animal Committee. Briefly, HepG2 cells (1 × 10^7^/0.2 ml PBS/mouse) were inoculated subcutaneously. Three days after the inoculation, the mice were randomly divided into five groups: the saline treated group (Control), **96** (0.2 mg/kg) group, **96** (1.0 mg/kg) treated group, **96** (5.0 mg/kg) treated group, and DOX (1.0 mg/kg) treated group. **96** were dissolved in saline containing 1% of DMSO and administered intraperitoneally every 2 d for 20 days. DOX was also administered intraperitoneally every 2 d for 20 d. The body weight and HepG2 xenografts volumes were recorded from the day of treatment, and xenografts volumes were calculated using the equation: tumour volume = length×(width)^2^×π/6. At the end of the experiment, mice were sacrificed and the xenografts were obtained for western blotting, HE staining and immunohistochemistry staining.

Western blotting was performed to detect the expression of Bcl-2, Bax and C-caspase-3 in HepG2 xenografts. Fresh HepG2 xenografts tissues were lysed and the obtained proteins were processed as the protein obtained from the HepG2 cells.

The HepG2 xenografts tissues were embedded using the paraffin, and sectioned for HE staining and immunohistochemistry staining. HE staining was performed routinely. The immunohistochemistry staining carried out as following. Briefly, the processed sections were incubated with the primary antibodies: Bcl-2 and TNF-α (NOVUS BIOLOGICALS) at 4 °C overnight. After washing, the anti-rabbit peroxidase-conjugated secondary antibody (Sigma-Aldrich, St. Louis, MO) was incubated for 1 h at RT. After washing with PBS, the cells were visualized using the 3,3'-diaminobenzidine (DAB) solution under the light microscope. The cell images were taken using a light microscopy (Olympus, Tokyo, Japan).

## Results and discussion

### Synthesis and structural characterization

**Scheme 1.**
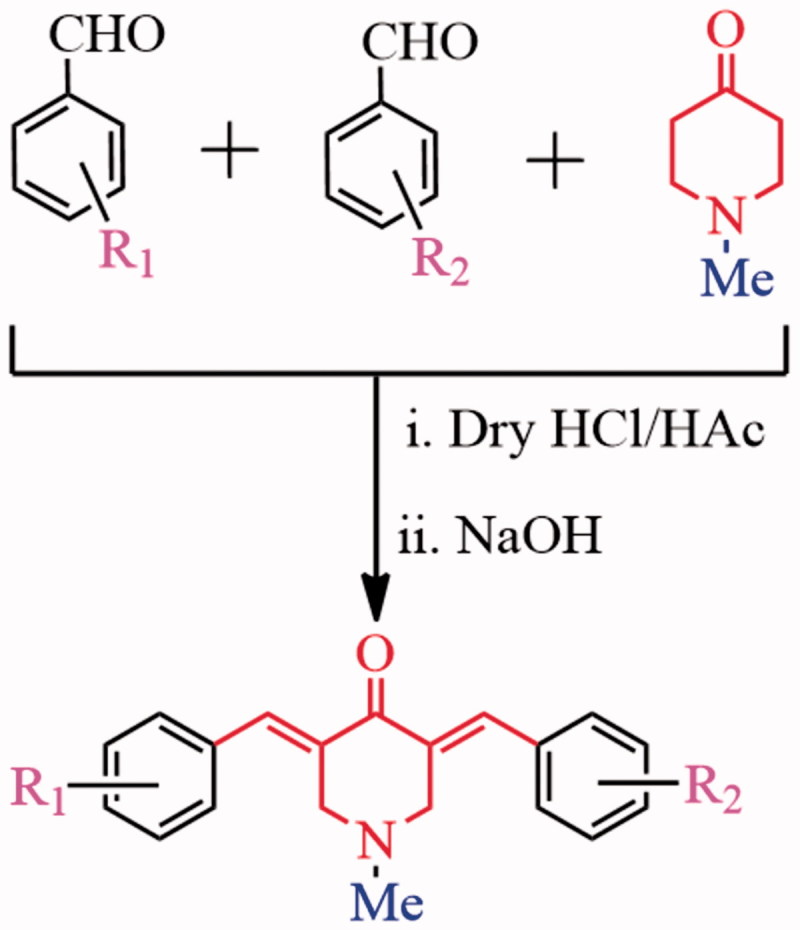
Synthetic strategy of BAPs **83**–**91**.

As shown in Scheme 1, BAPs **83–91** were synthesized by a step synthesis protocol of Claisen–Schmidt condensation^12^. *N*-Methyl-4-piperidinone and two aromatic aldehydes in 1:1:1 ratio interacted under the catalysis of dry HCl gas to generate three components, including two symmetric BAPs and a dissymmetric BAP. The dissymmetric BAP can be purified on silica gel by column. The yields of **83–91** can reach ca. 40%∼47%. Their structures were confirmed by ^1^H NMR, ^13^C NMR, IR and HRMS. BAPs **83–91** provide the different electronic environment in two sides of BAPs because there are dissymmetric structure characteristics in BAPs, which will cause significant differences in the NMR signals. For example, ^1^H NMR signals of –CH=C–C=O in two sides of BAPs split into two groups of unimodal signals, and chemical shifts are in the range of 8.24–7.69 ppm. Similarly, ^1^H NMR signals of –CH_2_ groups in central piperidone ring are also divided into two groups, which are in the range of 3.86–3.57 ppm. In ^13^C NMR spectra, the carbon atoms of –CH_2_ groups likewise appear in different chemical shifts (56.90–56.12 ppm). According to FTIR spectra, the characteristic bands of –C=O group are shown in around 1682–1663 cm^−1^. The strong bands of –C=C group in α,β-unsaturated ketone and aromatic ring are shown in around 1626–1577 cm^−1^.

**Scheme 2.**
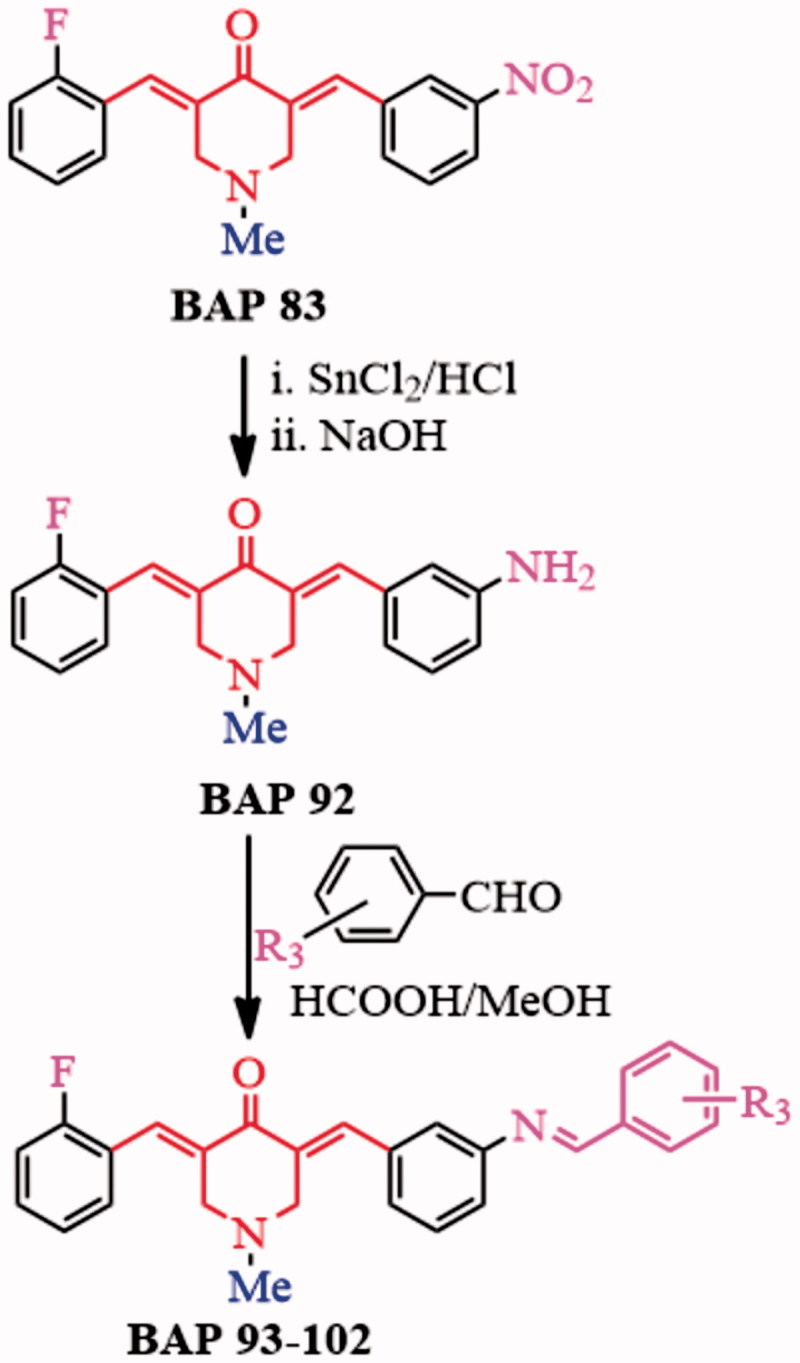
Synthetic strategy of BAPs **93**–**102**.

BAP **92** was obtained through nitro-reduction of **83** using stannous chloride according to a reported method^19^.The other aromatic aldehydes were following introduced into –NH_2_ fragment of **92** through Schiff-base condensation to generate BAPs **93–102** (Scheme 2), whose yields can reach ca. 71–88%. The structures, dissymmetric structure characteristics as well as different signals of BAPs **93–102** were confirmed by ^1^H NMR, ^13^C NMR, IR and HRMS. For example, the chemical shift of about 7.67–7.89 ppm should correspond to two groups of proton signals of –CH = C–C=O in two sides of BAPs. In addition, the single band in about 8.69–8.96 ppm appeared in the ^1^H NMR spectra, which proved the form of –CH=N bond. For **93**, **96**, **98–102**, *ortho-*hydroxyl-substituted aromatic aldehydes were grafted to Schiff-base BAPs, and the chemical shift of 13.49–12.08 ppm should correspond to *ortho-*hydroxyl proton from target BAPs. In BAPs **98–100**, other proton signals of hydroxyl also can be found in 9.78–8.53 ppm. Additionally, HRMS results displayed that the molecular weight are basically consistent with the calculated results, which further confirmed the correctness of their structures.

### Cytotoxicity and anti-inflammatory activity analysis of BAPs

The cytotoxicity activities against human carcinoma cell lines MCF-7, HCT116, A549, SGC7901, HepG2, HeLa, THP-1 and toxicity toward LO2 cell lines for BAPs were evaluated by MTT method. DOX and Curcumin were used as positive controls. The selectivity index (SI) is the quotient of the IC_50_ values towards LO2 cells. The results are shown in Table 1.

For dissymmetric **83–92**, IC_50_ values for about 26% of target BAPs against human carcinoma cell lines were lower than 5.0 μM, while especially for 2-F-substituted **83** and **92**, this ratio can reach about 86%. Structurally, **83** and **92** contain the electron-withdrawing 3-NO_2_group and electron-donating –NH_2_ substitutes, respectively. This is basically consistent with previous studies, which showed that dissymmetric BAPs containing electron-donating and electron-withdrawing groups demonstrated more potent anti-cancer activities^12^. More significantly, SI of **83** and **92** were greater than 10 for both HepG2 and THP-1. Especially for **92**, the IC_50_ values against HepG2 and THP-1 are only 1.1 μM and 1.2 μM, respectively, while SI can reach 17.2 and 15.8.

In order to improve the anti-cancer activities and reduce the toxicity, a series of Schiff-base substituted BAPs **93–102** were synthesized based on Schiff-base condensation reaction between **92** and aromatic aldehydes. The IC_50_ values of about 47% of target BAPs whose are lower than 5.0 μM. For HCT116 cell, IC_50_ values of 2-OH-4-F-substituted **93** and 2-OH-4-Br-substituted **96** are slightly lower than **92**. For A549 cell, IC_50_ values of **96** and 2,3,4-OH-substituted **100** are slightly lower than **92**. For MCF-7, SGC7901, HepG2, HeLa and THP-1, BAP **96** displayed distinctly lower IC_50_ value than that of **92** and the other Schiff-base substituted BAPs. In terms of SI of BAPs, SI for **96** against A549, HepG2 and THP-1 towards LO2 cells can reach 12.4, 19.9 and 18.1, which were slightly higher compared to **92**. Structural analysis displayed that R_3_ substituents, such as 2-OH-5-Br (**96**), 2-OH-5-F (**93**), and 2,3,4-OH (**100**), contributed to the improvement of anti-hepatoma activity. However, the other groups have little effect on the improvement of anti-hepatoma activity.

**Figure 2.**
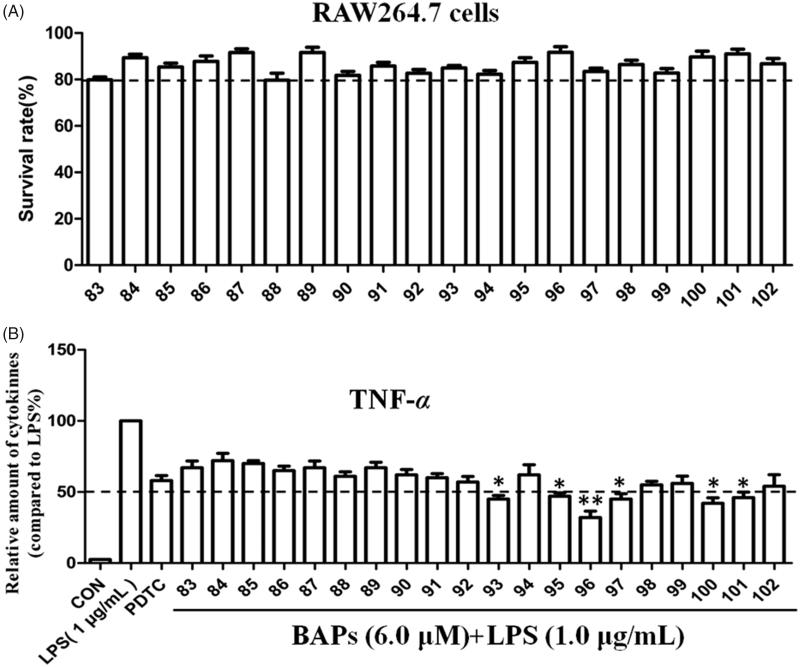
(A) The cytotoxicity of all BAPs against RAW264.7 cells by MTT assay *in vitro*. (B) The levels of TNF-α induced by LPS stimulation in RAW 264.7 cells through ELISA analysis. PDTC was used as a positive control. The results were presented as the percent of LPS control. Each bar represents the mean ± SD of three independent experiments. Statistical significance relative to the LPS group is indicated: ∗*p* < 0.05; ∗∗*p* < 0.01.

**Figure 3.**
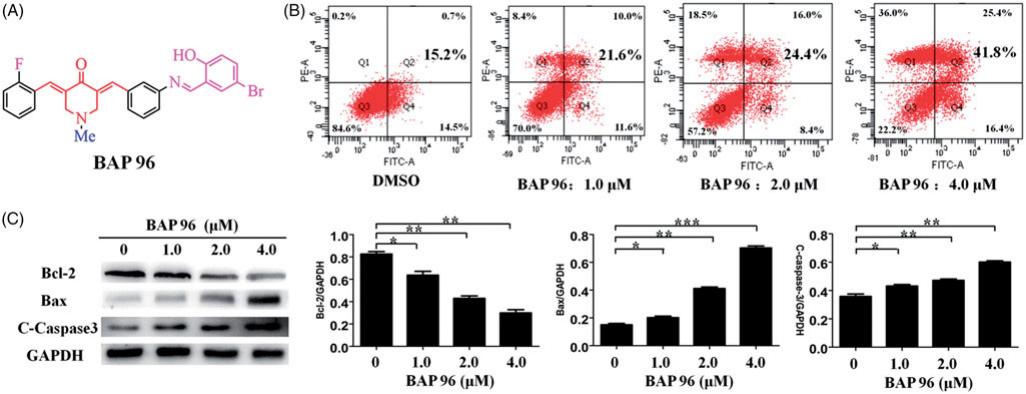
(A) The structures of **96**. (B) **96** induces HepG2 cells apoptosis. **p < 0.01, ***p < 0.001 versus the negative control. (C) The effect of **96** on the expression of Bax, Bcl-2, and C-caspase-3 detected by western blot. The data are representative of three independent experiments. *p < 0.05, **p < 0.01, ***p < 0.001 versus the negative control (one-way ANOVA followed by Dunnett’s test).

TNF-α is a common pro-inflammatory factors that can be produced by LPS-stimulated RAW264.7 macrophage^14^^,^^24^. In order to evaluate the anti-inflammatory activity of BAPs, the effect of BAPs on TNF-α production in LPS-stimulated RAW264.7 cells was detected by ELISA. As shown in Figure 2(A), the cell toxicity tested by MTT indicated that all BAPs were not significantly toxic to RAW264.7 cells at 6.0 μM. As shown in Figure 2(B), the anti-inflammatory results showed that some of BAPs had better anti-inflammatory activity than PDTC. In the presence of BAPs, the secretion of TNF-α was significantly decreased about 58% from LPS-stimulated RAW264.7 cells. For dissymmetric **83–92**, nine BAPs did not decrease the secretion of inflammatory cytokines compared with that of PDTC at concentration of 6.0 *μ*M. Only **92** presented slightly lower expression of TNF-*α* secretion (about 57%). After Schiff-base condensation reaction, **93** and **95–102** can inhibit the secretion of TNF-*α* and superior to PDTC group. Therein, BAPs **93**, **96**, **97**, and **100** significantly inhibited the TNF-*α* secretion, which is 45% lower than PDTC. Especially, **96** exhibited the strongest inhibitory effect against TNF-α expression. Based on the analysis of cytotoxicity and anti-inflammatory activity, the most potent **96** (Figure 3(A)) was selected for further biological assessment in this study.

### BAP 96 induced HepG2 cells apoptosis

To further investigate the underlying mechanism of decreased cell proliferation, the apoptosis mechanism of **96** on HepG2 cells was determined by flow cytometry. As shown in Figure 3(B), the results showed that **96** (2.0 μM) can induce 24.4% apoptosis of HepG2 cells. Under the concentration 4.0 μM of **96**, the apoptosis rate can reach about 41.8%. In addition, the results indicated that HepG2 cells showed a dose-dependent apoptosis after treatment with **96** for 24 h, including early and late apoptosis.

The effect of **96** on the expression levels of apoptosis-related proteins (Bcl-2, BAX, and C-Caspase-3) in HepG2 cells was determined by Western blot. The results are shown in Figure 3(C). After treatment with **96** (1.0, 2.0, and 4.0 μM), the expression of anti-apoptotic protein Bcl-2 was decreased in a dose-dependent manner. Meanwhile, the expression of pro-apoptotic protein BAX and C-caspase-3 were dose-dependently increased. **96** (4.0 μM) showed the most effective effects, which is consistent with pattern of the apoptosis rate. In conclusion, **96** could induce HepG2 cells apoptosis through up-regulating the expression of C-caspase-3 and Bax proteins and down-regulating the expression of Bcl-2 protein.

### BAP 96 inhibited NF-κB activation

**Figure 4.**
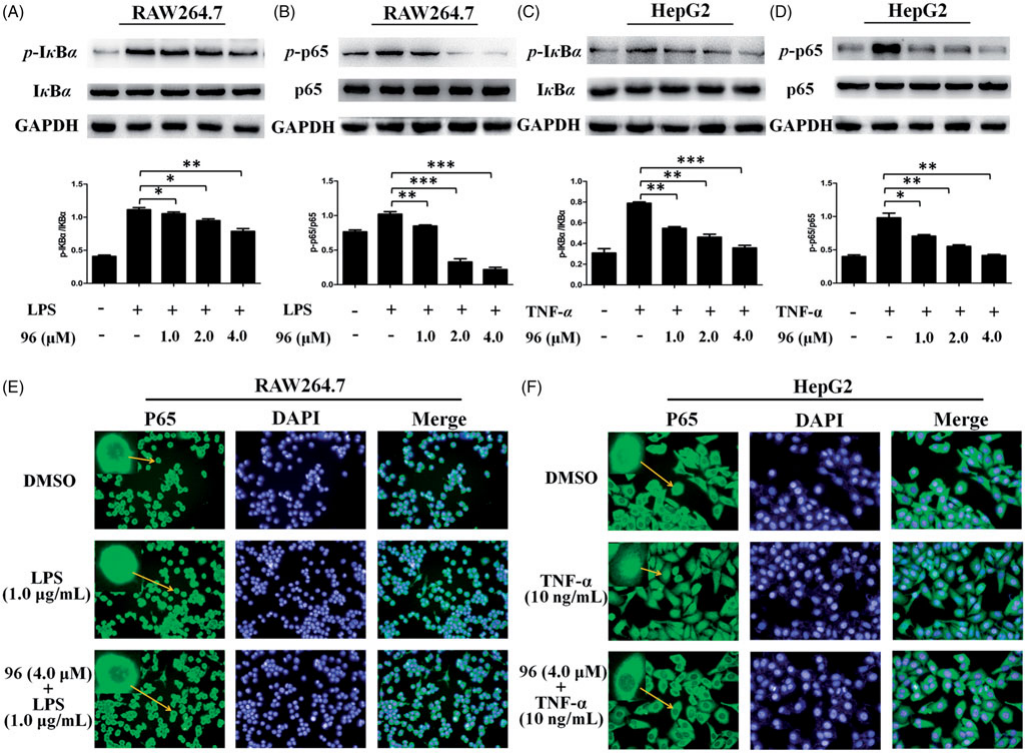
(A–D) Inhibitory effects of **96** on LPS-stimulated phosphorylation of I*κ*B*α* (A) and p65 (B) in RAW264.7 cells and TNF-*α*-stimulated phosphorylation of I*κ*B*α* (C) and p65 (D) in HepG2 cells, respectively. Data represent as the mean ± SD of triplicate tests. *p < 0.05, **p < 0.01 compared with the LPS or TNF-*α* alone group. (E, F) Effects of **96** on the nuclear translocation of NF-*κ*B p65 protein in the LPS-stimulated RAW264.7 cells (E) or TNF-*α*-stimulated HepG2 cells (F) by immunofluorescence assay. These experiments were repeated three times with similar results.

It is known that NF-*κ*B has been implicated in the crossroad of inflammation and cancer^25^^,^^26^. In our previous study, some BAPs (such as **23** and **67**) could inhibit NF-κB activation by inhibiting the phosphorylation of NF-κB (P65) and IκB^13^^,^^14^. Herein, it is an urgent to resolve whether **96** has the same mechanism of action as **23** and **67**. Therefore, the RAW264.7 cells were pretreated with LPS, and then treated with different concentrations (1.0, 2.0, 4.0 *μ*M) of **96**. As shown in Figure 4(A,B), the expression levels of LPS-induced *p*-IκBα and *p*-p65 were effectively decreased in a dose-dependent manner. Similarly, the HepG2 cells were pretreated with TNF-α, then treated with different concentrations (1.0, 2.0, and 4.0 μM) of **96**. As shown in Figure 4(C,D), the expression levels of the phosphorylation of IκBα and p65 protein in HepG2 cells induced by TNF-α showed in a dose-dependent downward trend. These results suggest that **96** can markedly inhibit the phosphorylation of IκBα and p65 in RAW264.7 and HepG2 cells to exhibit anti-hepatoma and anti-inflammatory activity.

### BAP 96 prevented the nuclear translocation of NF-κB induced by TNF-α or LPS

To evaluate the inhibitory effects of **96** against LPS or TNF-α-induced activation of NF-*κ*B, the p65 nuclear translocation in RAW264.7 and HepG2 cells were detected using immunofluorescence. DMSO was taken as a control. As shown in Figure 4(E,F), in control groups, majority of the p65 subunit were distributed in the cytoplasm of the RAW264.7 and HepG2 cells. After stimulated with 1.0 μg/mL of LPS or 10 ng/mL of TNF-α, many of p65 were transcribed into the cell nucleus of RAW264.7or HepG2 cells, but **96** (4.0 μM) prevented the p65 nuclear translocation and maintained p65 subunit mainly distributed in the cytoplasm of RAW264.7 cells or HepG2 cells, respectively. These results suggested that **96** could inhibit LPS or TNF-*α*-induced activation of NF-*κ*B through preventing the P65 nuclear translocation to exhibit potential anti-hepatma and anti-inflammatory activities.

### Molecular docking study

**Figure 5.**
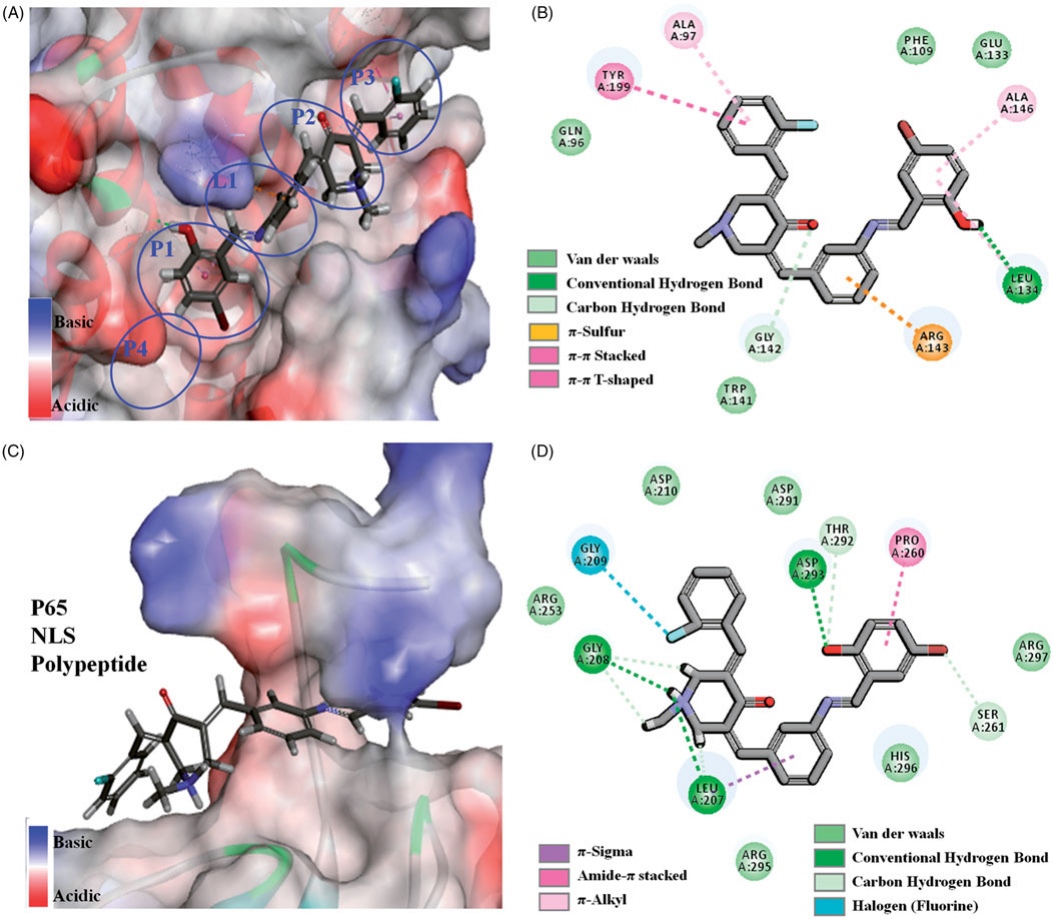
(A, B) 3D model and 2D model of the interaction between simulated **96** and the active site of Bcl-2 protein. (C, D) 3D model and 2D model of the interaction between simulated **96** with the active site of NF-*κ*B p65.

To further verify the interaction between **96** and Bcl-2, NF-κB/p65 proteins, C-DOCKER module in Discovery Studio 2017R2 was used as the molecular docking study^27^^,^^28^. The crystal structures of Bcl-2 (PDB1YSW) and NF-κB/p65 (PDB 1MY5) protein were optimized. For Bcl-2 protein, 5-bromo-2-hydroxybenzylidene inserts into the biggest P1 hydrophobic pocket by hydrophobic interaction (Figure 5(A,B)). There is strong hydrogen bond between hydroxyl and hinge residue of LEU A134. The central benzylidene crosses the narrow and shallow L1 channel by π-sulphur from ARG A143. The central piperidone and 2-fluorobenzylidene of **96** inserts into P2 and P3 hydrophobic pocket in turn. It is different from reported **23** and **67** that longer linear molecule of **96** can stretch and insert into P3 hydrophobic pocket of Bcl-2 protein^13^^,^^14^. For NF-*κ*B/p65, Schiff-base bridged 5-bromo-2-hydroxybenzylidene of **96** crosses the channel of p65 protein, in which there are hydrogen bonds and π–π stacking interactions (Figure 5(C,D)). In addition, the central piperidone and 2-fluorobenzylidene attach to the surface of the protein by hydrogen bonds and Halogen from amino acid residue. For crystal structures of Bcl-2, NF-κB/p65 proteins, the linear molecule is more suitable for anastomosis with the tubular cavity of Bcl-2 protein, while flexible or semi-rigid linear molecules are easier to go through the channel of p65 protein. It happens that simulated **96** is a semi-rigid linear molecule, and the aforementioned discussion proved simulated **96** could reasonably bind to the active site of Bcl-2 and NF-*κ*B/p65 protein. The results could verify the consistency between aforementioned western blot data and the data related to anti-hepatoma and anti-inflammatory activities of **96**.

### BAP 96 inhibited HepG2 xenografts growth *in vivo*

**Figure 6.**
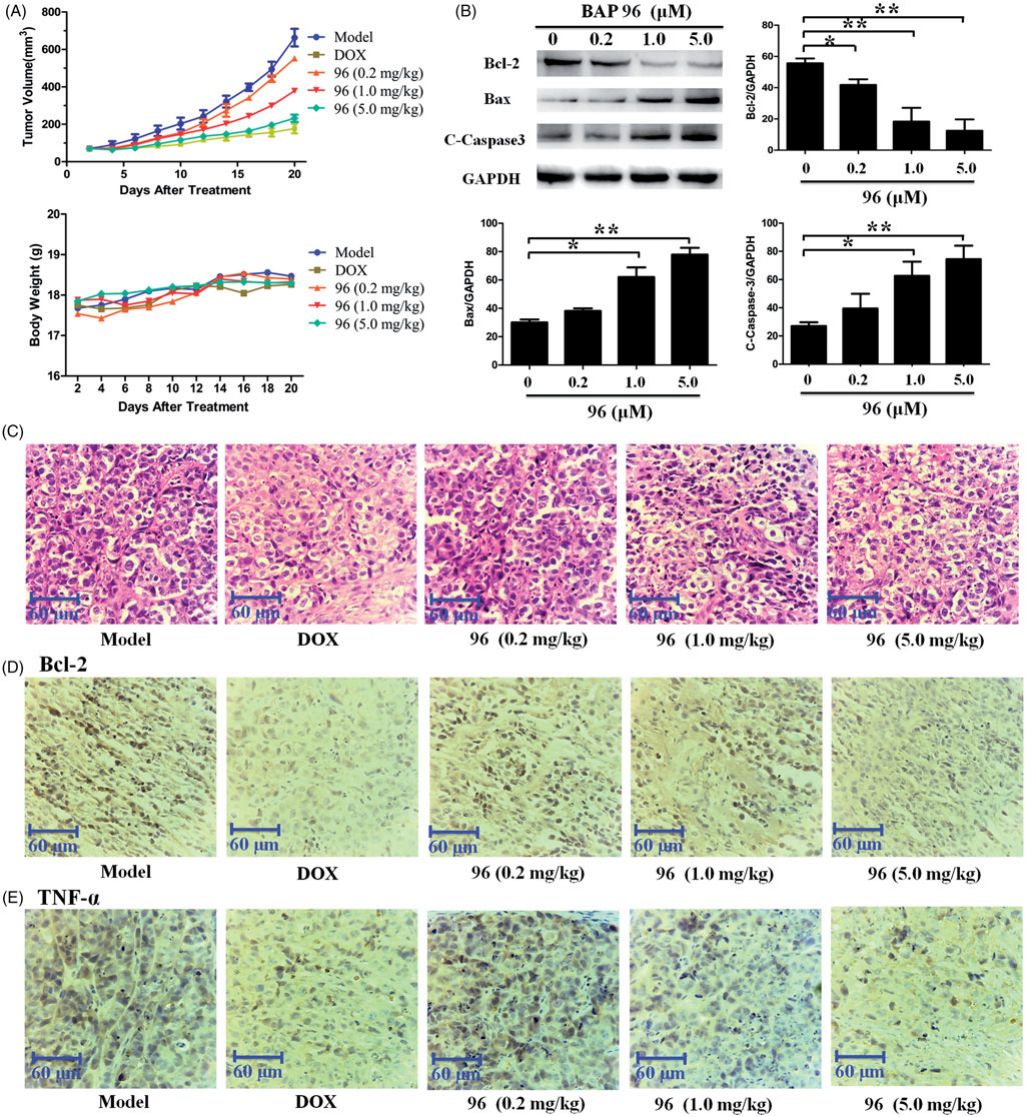
The inhibitory effects of BAP **96** on inhibiting HepG2 xenografts growth *in vivo*. (A) The HepG2 xenografts growth curves very two days and mice body weight curves every 2 d. (B) The expression of Bcl-2, Bax, and C-caspase-3 in the HepG2 xenografts of five groups. (C) The morphology of the HepG2 xenografts of the five groups detected by HE staining. (D) The expression of Bcl-2 and (E) the expression of TNF-α in the HepG2 xenografts of the five groups investigated by immunohistochemistry staining. Scale bar = 60 μm (C, D, E).

In order to detect the anti-hepatoma effect of **96** *in vivo*, the nude mice xenografts with HepG2 cells were established. The xenografts volume and body weight of nude mice were measured every 2 d. Twenty days after treatment with **96**, all xenografts were obtained and measured. As shown in Figure 6(A), 9**6** (0.2 mg/kg) and **96** (1.0 mg/kg) slightly decreased the growth of HepG2 xenografts; but **96** (5.0 mg/kg) significantly inhibited the growth of HepG2 xenografts as well as the positive drug DOX. However, the weight of all the mice increased until the end of the experiment, which showing that **96** is correspondingly nontoxic at the experimental doses. These results suggest that **96** could significantly inhibit the growth of HepG2 xenografts with nontoxic effect on the growth state of the mice.

Western blotting was used to determine the expression levels of apoptosis-related proteins (Bcl-2, BAX, and C-Caspase-3) in HepG2 xenografts. As shown in Figure 6(B), 9**6** (0.2, 1.0 and 5.0 mg/kg) dose-dependently decreased the expression of anti-apoptotic protein Bcl-2. Meanwhile, the expression of pro-apoptotic protein BAX and C-caspase-3 were significantly increased in a dose-dependent manner by **96**. Collectively, **96** (5.0 mg/kg) showed the most effective effects in inducing the apoptosis of the HepG2 cells.

Haematoxylin and eosin (HE) staining was performed to investigate the histopathological changes. As shown in Figure 6(C), **96** and DOX made the HepG2 cell nucleus wrinkled, which was completely different from that of smooth HepG2 cell nucleus in model group. Most obviously, a plenty of fracted cells were detected on **96** (5.0 mg/kg)- and DOX-treated groups. These results suggest that the anti-hepatoma effect of **96** (5.0 mg/kg) is considerable to the positive drug DOX *in vivo*.

Furthermore, immunohistochemistry staining of Bcl-2 and TNF-α in the HepG2 xenografts were carried out to evaluate the anti-hepatoma and anti-inflammatory activities. As shown in Figure 6(D,E), both Bcl-2 and TNF-α were obviously expressed in the cytoplasm of HepG2 cells in model group, but were significantly reduced in DOX- and **96** (5.0 mg/kg)-treated groups. Therefore, we get the conclusion that **96** possess both the anti-hepatoma and anti-inflammation capabilities.

## Conclusions

In this study, a series of dissymmetric BAPs **83–102** were synthesized and characterized, as well as evaluated their anti-chepatoma and anti-inflammatory activities. The 5-bromo-2-hydroxybenzylidene and 2-fluorobenzylidene substituted **96** showed the best bioactivities with IC_50_ values lower than 2.0 μM against experimental HepG2, A549, and THP-1 cells; The SI values can reach 12.4, 19.9, and 18.1, which proves lower toxicity toward LO2 cells. In addition, **96** could induce HepG2 cells apoptosis through up-regulating the expression of C-caspase-3 and Bax proteins, down-regulating the expression of Bcl-2 protein. Furthermore, **96** can markedly inhibit LPS or TNF-*α*-induced activation of NF-*κ*B through both inhibiting the phosphorylation of IκBα and p65 and preventing the P65 nuclear translocation to exhibit anti-hepatoma and anti-inflammatory activities. This mechanism of action was also proved by the Molecular docking between simulated **96** and Bcl-2, NF-κB/p65 proteins. Moreover, **96** inhibited HepG2 xenografts growth in *vivo.* In conclusion, **96** possess both the anti-hepatoma and anti-inflammation capabilities, and could be developed as a potential multifunctional agent for treatment of inflammatory diseases and cancers.

## Supplementary Material

Supplemental Material
