# Supplementary material for: Potential multifunctional agents with anti-hepatoma and anti-inflammation properties by inhibiting NF-кB activation
Source: J Enzyme Inhib Med Chem. 2019 Jul 10;34(1):1287–97. doi: 10.1080/14756366.2019.1635124 (PMC6691761; doi:10.1080/14756366.2019.1635124)
Supplement: Supplemental Material [file IENZ_A_1635124_SM5573.pdf]

Supporting Information

Potential multifunctional agents with anti-hepatoma and anti-inflammation properties by inhibiting NF-κB activation

Chang-Ming Su[1], Gui-Ge Hou[1], Chun-Hua Wang[1]*, Hong-Qin Zhang[2], Cheng Yang[2]*, Mei Liu[3],Yun Hou[2]*

[1]School of Pharmacy, the Key Laboratory of Prescription Effect and Clinical Evaluation of State Administration of Traditional Chinese Medicine of China, Binzhou Medical University, Yantai, 264003, P. R. China; [2]School of Basic Medical Sciences, Binzhou Medical University, Yantai, Shandong, 264003, P. R. China; and [3]The Second People's Hospital of Dongying, Dongying, Shandong, 257000, P. R. China

Tel: +86-535-6913317; fax: +86-535-6913718;

**E-mail address: houyun820424@163.com (Y. Hou), chunhuawang508@126.com (C.-H. Wang), yangchxn@163.com (C. Yang)*

Structural characterization

BAP 84: Light yellow powder; Yield: 41%; m.p: 170-172℃; IR (cm^{-1}): 2950 (m), 2860(m), 2792(m), 1677(s), 1617(s), 1590(s), 1522(s), 1496(s), 1348(s), 12 96(s),1180(s), 1102(s), 1057(s), 967(s), 921(s), 841(s), 805(s), 743(s). ^1H NMR (400 MHz, $CDCl_3$) δ 8.26 (s, 1H, -C=CH), 8.24 (s, 1H, -C=CH), 7.84 (d, J = 10.6 Hz, 2H, $-C_6H_4$), 7.72 (d, J = 7.6 Hz, 1H, $-C_6H_4$), 7.64 (t, J = 8.1 Hz, 1H, $-C_6H_4$), 7.36 – 7.29 (m, 2H, $-C_6H_3$), 7.03 – 6.86 (m, 1H, $-C_6H_3$), 3.78 (s, 2H, $-CH_2$), 3.65 (s, 2H, $-CH_2$), 2.47 (s, 3H, $-NCH_3$). ^{13}C NMR (150 MHz, C DCl_3) δ 184.82, 162.44 (dd, J = 253.4, 12.4 Hz), 160.26 (dd, J = 255.8, 12.8 Hz), 147.32, 135.73, 135.00, 134.23, 133.23, 132.64, 130.63 (dd, J = 9.7, 4.0 Hz), 128.66, 127.67 (d, J = 2.9 Hz), 123.36, 122.50, 118.28 (dd, J = 13.8, 3. 9 Hz), 110.53 (dd, J = 21.3, 3.8 Hz), 103.54, 55.77, 44.76, 28.67. HREI-MS: M^+ calcd for $C_{20}H_{16}F_2N_2O_3$ 370.1129, found 370.1132.

BAP 85: Light yellow powder; Yield: 43%; m.p: 175-177℃; IR (cm^{-1}): 3

082(m), 2935(m), 2781(m), 1677(s), 1617(s), 1591(s), 1529(s), 1437(s), 1271(s), 1188(s), 1113(s), 995(s), 929(s), 870(s), 834(s), 783(s). ^{1}H NMR (400 MHz, $CDCl_3$) δ 8.26 (s, 1H, -C=CH), 8.24 (s, 1H, -C=CH), 7.82 (s, 1H, $-C_6H_4$), 7.7 3 – 7.68 (m, 2H, $-C_6H_4$), 7.64 (t, *J* = 7.9 Hz, 1H, $-C_6H_4$), 6.92 (d, *J* = 6.3 H z, 2H, $-C_6H_3$), 6.85 (t, *J* = 8.7 Hz, 1H, $-C_6H_3$), 3.78 (s, 2H, $-CH_2$), 3.74 (s, 2 H, $-CH_2$), 2.51 (s, 3H, $-NCH_3$). ^{13}C NMR (100 MHz, $CDCl_3$) δ 185.99, 162.8 8 (dd, *J* = 249.4, 12.9 Hz), 148.35, 137.94 (t, *J* = 9.5 Hz), 136.60, 135.90, 1 35.13, 134.65, 134.18 (t, *J* = 2.6 Hz), 133.62, 129.64, 124.35, 123.53, 112.85, (dd, *J* = 19.2Hz, 12.1Hz), 104.49 (t, *J* = 25.4 Hz), 56.70, 56.57, 45.81. HREI-MS: M^+ calcd for $C_{20}H_{16}F_2N_2O_3$ 370.1129, found 370.1135.

BAP 86: Light yellow powder; Yield: 45%; m.p: 173-175℃; IR (cm^{-1}): 2 950(m), 2848(m), 1682(s), 1626(s), 1597(s), 1523(s), 1483(s), 1348(s), 1306(s), 1181(s), 1096(s), 1057(s), 998(s), 932(s), 877(s), 821(s), 736(s). ^{1}H NMR (400 MHz, $CDCl_3$) δ 8.25 (s, 1H, -C=CH), 8.23 (s, 1H, -C=CH), 7.81 (s, 2H, $-C_6H_4$), 7.71 (d, *J* = 7.6 Hz, 1H, $-C_6H_4$), 7.64 (t, *J* = 8.2 Hz, 1H, $-C_6H_4$), 7.17 – 7.05 (m, 2H, $-C_6H_3$), 7.02 (t, *J* = 6.9 Hz, 1H, $-C_6H_3$), 3.77 (s, 2H, $-CH_2$), 3.6 8 (s, 2H, $-CH_2$), 2.49 (s, 3H, $-NCH_3$). ^{13}C NMR (100 MHz, $CDCl_3$) δ 185.76, 158.76 (dd, *J* = 121.0, 2.1 Hz), 156.32 (dd, *J* = 125.7, 2.1 Hz), 148.29, 136. 62, 136.01, 135.24, 135.09, 133.82, 129.67, 128.36 (dd, *J* = 3.2, 1.7 Hz), 123. 96 (d, *J* = 82.7 Hz), 56.73, 56.62, 45.78. HREI-MS: M^+ calcd for $C_{20}H_{16}F_2N_2O_3$ 370.1129, found 370.1131.

BAP 87: Light yellow powder; Yield: 47%; m.p: 190-192℃; IR(cm^{-1}): 29 47(m), 2846(m), 2780(m), 1677(s), 1607(s), 1527(s), 1487(s), 1344(s), 1271(s), 1185(s), 1095(s), 919(s), 826(s), 742(s). ^{1}H NMR (400MHz, $CDCl_3$) δ 8.25(s, 1H, $-C_6H_4$), 8.23(s, 1H, $-C_6H_4$), 7.90 (s, 1H, -C=CH), 7.85 (s, 1H, -C=CH), 7. 71 (d, *J* = 7.6Hz, 1H, $-C_6H_4$), 7.64 (t, *J* = 7.8Hz, 1H, $-C_6H_4$), 7.43 (dd, *J* = 8.1, 2.1Hz, 1H, $-C_6H_3$), 7.26–7.18 (m, 1H, $-C_6H_3$), 7.11 (td, *J* = 8.2, 2.4Hz, 1 H, $-C_6H_3$), 3.80 (s, 2H, $-CH_2$), 3.62 (s, 2H, $-CH_2$), 2.45 (s, 3H, $-NCH_3$). ^{13}C NMR (100 MHz, $CDCl_3$) δ 185.63, 163.59, 161.06, 148.28, 136.58, 136.02, 13 5.61, 134.16, 131.34, 131.25, 129.70, 125.76, 125.66, 124.36, 123.98 (d, *J* = 7

6.6Hz), 120.82, 120.57, 114.53 (d, J = 21.4Hz), 56.65, 56.12, 45.50. HREI-MS: M^+ calcd for $C_{20}H_{16}BrFN_2O_3$ 430.0328, found 430.0323.

BAP 88: Light yellow powder; Yield: 40%; m.p: 154-156℃; IR (cm^{-1}): 2 947(m), 2847(m), 2785(m), 1668(s), 1617(s), 1577(s), 1506(s), 1480(m), 1416(s), 1380(s), 1335(s), 1270(s), 1180(s), 1122(s), 996(s), 918(s), 883(s). ^{1}H NMR (4 00 MHz, $CDCl_3$) δ 7.86 (s, 1H, -C=CH), 7.78 (s, 1H, -C=CH), 7.42 (dd, J = 8.1, 2.1 Hz, 1H, $-C_6H_3$), 7.24 – 7.19 (m, 1H, $-C_6H_3$), 7.09 (td, J = 8.3, 2.1 H z, 1H, $-C_6H_3$), 6.63 (s, 2H, $-C_6H_2$), 3.91 (s, 3H, $-OCH_3$), 3.90 (s, 6H, $-OCH_3$), 3.80 (s, 2H, $-CH_2$), 3.57 (s, 2H, $-CH_2$), 2.43 (s, 3H, $-NCH_3$). ^{13}C NMR (100 MHz, $CDCl_3$) δ 186.13, 162.16 (d, J = 253.8 Hz), 153.06, 139.18, 137.47, 1 34.58, 134.18, 132.05, 131.62 (d, J = 3.7 Hz), 131.26 (d, J = 8.6 Hz), 130.57, 125.57 (d, J = 9.5 Hz), 120.56 (d, J = 24.4 Hz), 114.41 (d, J = 21.3 Hz), 1 07.88, 60.95, 57.15, 56.21, 56.17, 45.54. HREI-MS: M^+ calcd for $C_{23}H_{23}BrFN O_4$ 475.0794, found 475.0790.

BAP 89: Light yellow powder; Yield: 47%; m.p: 167-169℃; IR (cm^{-1}): 3 000(m), 2843(m), 1673(s), 1611(s), 1578(s), 1506(s), 1457(m), 1414(s), 1267(s), 1234(s), 1122(s), 1018(s), 1001(s), 831(s), 762(s). ^{1}H NMR (400 MHz, $CDCl_3$) δ 8.08 (s, 1H, -C=CH), 7.76 (s, 1H, -C=CH), 7.36 (t, J = 7.6 Hz, 1H, $-C_6H_4$), 7.21 (d, J = 7.4 Hz, 1H, $-C_6H_4$), 6.99 (t, J = 7.5 Hz, 1H, $-C_6H_4$), 6.94 (d, J = 8.3 Hz, 1H, $-C_6H_4$), 6.63 (s, 2H, $-C_6H_2$), 3.91 (s, 3H, $-OCH_3$), 3.90 (s, 6 H, $-OCH_3$), 3.87 (s, 3H, $-OCH_3$), 3.80 (s, 2H, $-CH_2$), 3.69 (s, 2H, $-CH_2$), 2.44 (s, 3H, $-NCH_3$). ^{13}C NMR (100 MHz, $CDCl_3$) δ 186.69, 158.44, 153.01, 138. 91, 136.52, 132.73, 132.64, 132.57, 130.88, 130.56, 130.23, 124.24, 119.98, 11 0.72, 107.76, 60.94, 57.22, 56.86, 56.18, 55.43, 45.58. HREI-MS: M^+ calcd for $C_{24}H_{27}NO_5$ 409.1889, found 409.1895.

BAP 90: Light yellow powder; Yield: 41%; m.p: 178-180℃; IR (cm^{-1}): 2 943(m),2836(m), 2777(m), 1663(s), 1610(s), 1577(s), 1504(s), 1450(m), 1416(s), 1324(s), 1270(s), 1251(m), 1165(s), 1124(s), 1065(m), 1022(s), 998(s), 932(s), 849(s), 826(m), 782(s). ^{1}H NMR (400 MHz, $CDCl_3$) δ 7.79 (s, 1H, -C=CH), 7. 75 (s, 1H, -C=CH), 7.39 (d, J = 8.6 Hz, 2H, $-C_6H_4$), 6.96 (d, J = 8.5 Hz, 2

H, -C_6H_4), 6.63 (s, 2H, -C_6H_2), 3.90 (s, 3H, -OCH_3), 3.90 (s, 6H, -OCH_3), 3.8 6 (s, 3H, -OCH_3), 3.79 (s, 4H, -CH_2), 2.49 (s, 3H, -NCH_3). ^{13}C NMR (100 M Hz, $CDCl_3$) δ 186.66, 160.31, 153.02, 138.90, 136.44, 136.18, 132.61, 132.37, 130.98, 130.83, 127.85, 114.08, 107.72, 60.94, 57.18, 56.90, 56.19, 55.32, 45.7 6. HREI-MS: M^+ calcd for $C_{24}H_{27}NO_5$ 409.1889, found 409.1883.

BAP 91: Light yellow powder; Yield: 42%; m.p: 164-166℃; IR (cm^{-1}): 2 924(m), 2853(s), 2776(m), 1677(s), 1587(s), 1509(s), 1461(m), 1309(m), 1257(s), 1160(s), 1128(m), 1025(s), 986(s), 916(s), 824(s), 782(m). ^{1}H NMR (400 MHz, $CDCl_3$) δ 7.80 (s, 1H), 7.69 (s, 1H), 7.54 –7.46 (m, 2H), 7.40 (d, *J* = 8.7 H z, 2H), 7.23 (d, *J* = 8.2 Hz, 1H), 6.98 (d, *J* = 8.7 Hz, 2H), 3.88 (s, 3H), 3.8 0 (s, 2H), 3.72 (s, 2H), 2.51 (s, 3H). ^{13}C NMR (100 MHz, $CDCl_3$) δ 186.68, 161.20, 153.18, 152.90, 138.27, 137.12, 136.38, 135.81, 132.32, 132.18, 131.87, 130.57, 127.85, 114.38, 108.28, 60.92,56.90, 56.49, 45.66. HREI-MS: M^+ calcd for $C_{21}H_{19}Cl_2NO_2$ 387.0793, found 387.0785.

BAP 93: Light yellow powder; Yield: 74%; m.p: 143-145℃; IR(cm^{-1}): 30 89(m), 2979(m), 2946(m), 2839(m), 1670(s), 1617(s), 1572(s), 1486(s), 1353(m), 1275(s), 1183(s), 1057(m), 859(m), 799(s), 759(s). ^{1}H NMR (400 MHz, DMS O) δ 12.51 (s, 1H, -OH), 8.96 (s, 1H, -N=CH), 7.69 (s, 1H, -C=CH), 7.68 (s, 1H, -C=CH), 7.60 – 7.49 (m, 4H, -C_6H_4), 7.46 (t, 3H, -C_6H_4), 7.36 – 7.28 (m, 3H, -C_6H_3), 7.00 (dd, 1H, -C_6H_3), 3.79 (s, 2H, -CH_2), 3.65 (s, 2H, -CH_2), 2.37 (s, 3H, -NCH_3). ^{13}C NMR (100 MHz, DMSO) δ 186.49, 163.17, 160.83 (d, *J* = 249.1 Hz), 156.82, 155.38 (d, *J* = 235.3 Hz), 149.12, 136.25, 135.76 135.18, 134.50, 131.97 (d, *J* = 8.6 Hz), 131.32, 130.29, 129.13, 127.02 (d, *J* = 4.7 Hz), 125.11 (d, *J* = 3.4 Hz), 123.79, 122.74, 122.62, 120.86 (d, *J* = 2 3.6 Hz), 120.08 (d, *J* = 7.6 Hz), 118.48 (d, *J* = 7.6 Hz), 117.28 (d, *J* = 23.4 Hz), 116.20 (d, *J* = 21.7 Hz), 56.77, 56.48, 45.61. HREI-MS: M^+calcd for $C_{27}H_{22}F_2N_2O_2$ 444.1649, found 444.1640.

BAP 94: Light yellow powder; Yield: 77%; m.p: 168-170℃; IR (cm^{-1}): 3 049(m), 2968(m), 1671(s), 1620(s), 1589(m), 1566(s), 1482(s), 1453(s), 1406(s), 1291(s), 1272(s), 1230(s), 1217(s), 1178(s), 1131(s), 1100(s), 1086(s), 1053(s),

1035(s), 978(s), 931(s), 914(s), 882(s), 842(s), 827(s), 807(s), 790(s), 754(s), 710(s). ^{1}H NMR (400 MHz, DMSO) δ 8.69 (s, 1H, -N=CH), 7.98 (s,1H, -C_6H_4), 7.96 (s, 1H, -C_6H_4), 7.67 (s, 1H, -C=CH), 7.67 (s, 1H, -C=CH), 7.61 (d, *J* = 8.1 Hz, 2H, -C_6H_4), 7.50 (dt, *J* = 20.4, 7.7 Hz, 3H, -C_6H_4), 7.39 (d, *J* = 9.9 Hz, 2H, -C_6H_4), 7.36 – 7.28 (m, 3H, -C_6H_4), 3.78 (s, 2H, -CH_2), 3.64 (s, 2H, -CH_2), 2.37 (s, 3H, -NCH_3). ^{13}C NMR (150 MHz, DMSO) δ 186.57, 163.38, 160.88 (d, *J* = 249.2 Hz), 150.04 (d, *J* = 19.2 Hz), 148.68, 136.34, 135.83, 135.21, 134.61, 132.06 (d, *J* = 9.2 Hz), 131.38 (d, *J* = 3.2 Hz), 130.38, 129.30, 125.58, 125.19, 123.79, 122.80, 122.18 (d, *J* = 9.5 Hz), 120.86, 118.33, 116.26 (d, *J* = 21.6 Hz), 109.871, 56.79, 56.52, 45.64. HREI-MS: M^+ calcd for $C_{27}H_{22}ClFN_2O$ 444.1405, found 444.1423.

BAP 95: Light yellow powder; Yield: 71%; m.p: 90-92℃; IR (cm^{-1}): 2946(m), 1669(s), 1616(s), 1575(s), 1553(s), 1483(s), 1452(s), 1350(s), 1331(s), 1299(s), 1272(s), 1231(s), 1215(s), 1180(s), 1132(s), 1101(s), 1052(s), 1032(s), 992(s), 915(s), 891(s), 821(s), 790(s), 758(s), 734(s). ^{1}HNMR (400MHz, DMSO) δ 8.69 (s, 1H, -N=CH), 8.15 (s, 1H, -C_6H_3), 7.93 (d, *J* = 7.8Hz, 1H, -C_6H_3), 7.81 (d, *J* = 8.0Hz, 1H, -C_6H_3), 7.68 (s, 1H, -C=CH), 7.67 (s, 1H, -C=CH), 7.67–7.52 (m, 3H, -C_6H_4), 7.44–7.25 (m, 5H, -C_6H_4), 3.78 (s, 2H, -CH_2), 3.64 (s, 2H, -CH_2), 2.37(s, 3H, -NCH_3). ^{13}CNMR (100 MHz, DMSO) δ 186.48, 160.83 (d, *J* = 250.5 Hz), 159.86, 151.49, 136.45 (d, *J* = 78.7 Hz), 135.76, 135.35, 134.43, 134.32, 132.22, 131.97 (d, *J* = 8.5 Hz), 131.66, 131.30 (d, *J* = 1.4 Hz), 130.66, 130.13, 128.89, 128.79, 127.00, 126.96, 125.11 (d, *J* = 3.3 Hz), 123.49, 122.69 (d, *J* = 12.8 Hz), 122.50, 116.20 (d, *J* = 21.8 Hz), 56.82, 56.48, 45.64. HREI-MS: M^+ calcd for $C_{27}H_{21}Cl_2FN_2O$ 478.1015, found 478.1010.

BAP 96: Light yellow powder; Yield: 78%; mp:104-106℃; IR (cm^{-1}): 3071(m), 2950(m), 2794(m), 1674(s), 1620(s), 1595(s), 1559(m), 1476(s), 1351(m), 1272(s), 1226(s), 1177(s), 1149(m), 985(s), 922(s), 920(s), 814(s), 785(s), 761(s). ^{1}H NMR (400 MHz, DMSO) δ 12.84 (s, 1H), 8.96 (s, 1H), 7.89 (s, 1H), 7.68 (s, 1H), 7.67 (s, 1H), 7.56 (d, *J* = 7.2 Hz, 2H), 7.51 (s, 2H), 7.46 (m, 3H), 7.33 (m, 1H), 6.97 (d, *J* = 8.8 Hz, 2H), 3.79 (s, 2H), 3.66 (s, 2H), 2.38

(s, 3H). ^{13}C NMR (100 MHz, DMSO) δ 186.46, 163.51, 163.02, 160.83 (d, *J* = 250.48 Hz), 159.67, 148.93, 136.25, 136.11, 135.72, 135.19, 134.48, 134.35, 131.99 (d, *J* = 8.6 Hz), 131.32 (d, *J* = 1.8 Hz), 130.30, 129.23, 127.08 (d, *J* = 4.4 Hz), 125.12 (d, *J* = 3.4 Hz), 123.79, 122.80, 121.68, 119.53, 116.20 (d, *J* = 21.7 Hz), 110.41, 56.73, 56.45, 45.59. HREI-MS: M^+ calcd for $C_{27}H_{22}BrF N_2O_2$ 504.0849, found 504.0845.

BAP 97: Light yellow powder; Yield: 79%; m.p: 156-158℃; IR (cm^{-1}): 2 955(m), 1674(s), 1619(s), 1579(s), 1534(s), 1485(s), 1453(s), 1347(s), 1271(s), 1245(s), 1224(s), 1186(s), 1107(s), 1069(s), 990(s), 952(s), 935(s), 918(s), 888 (s), 838(s), 823(s), 806(s), 783(s), 761(s), 741(s). ^1H NMR (400 MHz, $CDCl_3$) δ 9.11 (dd, *J* = 5.7, 2.7 Hz, 1H, -C_6H_3), 8.77 (s, 1H, -N=CH), 8.51 – 8.26 (m, 1H, -C_6H_3), 7.89 (s, 1H, -C=CH), 7.84 (s, 1H, -C=CH), 7.48 (t, *J* = 8.0 Hz, 1H, -C_6H_3), 7.39 – 7.25 (m, 6H, -C_6H_4), 7.18 (t, *J* = 7.4 Hz, 1H, -C_6H_4), 7.15 – 7.06 (m, 1H, -C_6H_4), 3.83 (s, 2H, -CH_2), 3.67 (s, 2H, -CH_2), 2.46 (s, 3H, -NCH_3). ^{13}C NMR (100 MHz, DMSO) δ 186.52, 163.21, 160.79 (d, *J* = 248.2 Hz), 156.80, 155.98 (d, *J* = 245.3 Hz), 149.32, 136.58, 135.56, 135.12, 134.40, 131.99 (d, *J* = 8.4 Hz), 131.31, 130.65, 129.34, 127.28 (d, *J* = 4.6 Hz), 125.21 (d, *J* = 3.6 Hz), 123.70, 122.71, 122.56, 120.67 (d, *J* = 21.4 Hz), 120.26 (d, *J* = 7.5 Hz), 118.48 (d, *J* = 7.4 Hz), 117.27 (d, *J* = 22.5 Hz), 11 6.39 (d, *J* = 21.1 Hz), 56.74, 56.40, 45.67. HREI-MS: M^+ calcd for $C_{27}H_{21}F_2N_3O_3$ 473.1551, found 473.1548.

BAP 98: Light yellow powder; Yield: 78%; m.p: 176-178℃; IR(cm^{-1}): 345 4(m), 3066(m), 2939(m), 1668(s), 1608(s), 1564(s), 1485(s), 1463(m), 1362(s), 1280(m), 1237(s), 1184(s), 1035(m), 934(s), 784(s), 750(s), 732(s). ^1H NMR (4 00 MHz, DMSO) δ 13.01 (s, 1H, -OH), 9.25 (s, 1H, -OH), 8.96 (s, 1H, -N=C H), 7.69 (s, 1H, -C=CH), 7.68 (s, 1H, -C=CH), 7.56 (dd, *J* = 14.7, 6.9 Hz, 2 H, -C_6H_4), 7.51 – 7.42 (m, 4H, -C_6H_4), 7.32 (dd, *J* = 14.9, 7.8 Hz, 2H, -C_6H_4), 7.11 (d, *J* = 7.4 Hz, 1H, -C_6H_3), 6.96 (d, *J* = 7.6 Hz, 1H, -C_6H_3), 6.81 (t, *J* = 7.8 Hz, 1H, -C_6H_3), 3.79 (s, 2H, -CH_2), 3.65 (s, 2H, -CH_2), 2.37 (s, 3H, -NCH_3). ^{13}C NMR (100 MHz, DMSO) δ 186.50, 165.20, 160.83 (d, *J* = 249.

0 Hz), 149.67, 148.84, 146.06, 136.25, 135.77, 135.26, 134.46, 131.98 (d, *J* = 8.9 Hz), 131.32 (d, *J* = 1.9 Hz), 130.28, 128.92, 127.02 (d, *J* = 4.4 Hz), 125. 12 (d, *J* = 3.4 Hz), 123.77, 123.28, 122.75, 122.62, 119.80, 119.56, 119.29, 11 6.21 (d, *J* = 21.6 Hz), 56.78, 56.48, 45.61. HREI-MS: M^+ calcd for $C_{27}H_{23}FN_2O_3$ 442.1693, found 442.1676.

BAP 99: Light yellow powder; Yield: 81%; m.p: 228-230℃; IR (cm^{-1}): 30 79(m), 3032(m), 2947(m), 1682(s), 1564(s), 1483(s), 1452(m), 1292(s), 1222(s), 1180(s), 1102(s), 983(m), 874(m), 757(s). ^1H NMR (400 MHz, DMSO) δ 12.0 8 (s, 1H, -OH), 9.14 (s, 1H, -OH), 8.88 (s, 1H, -N=CH), 7.68 (s, 1H, -C=C H),7.67 (s, 1H, -C=CH), 7.63–7.37 (m, 7H, $-C_6H_4$), 7.32 (dd, *J* = 14.9, 7.8 Hz, 2H, $-C_6H_4$), 7.06 (d, *J* = 2.4 Hz, 1H, $-C_6H_3$), 6.88 (dd, *J* = 8.7, 2.5 Hz, 1H, $-C_6H_3$), 6.81 (d, *J* = 8.8 Hz, 1H, $-C_6H_3$), 3.79 (s, 2H, $-CH_2$), 3.65 (s, 2H, $-CH_2$), 2.37(s, 3H, $-NCH_3$). ^{13}C NMR (100 MHz, DMSO) δ 186.48, 164.35, 163. 52, 160.83 (d, *J* = 249.0 Hz), 153.47, 150.09, 149.45, 136.19, 135.75, 135.35, 134.37, 131.97 (d, *J* = 8.8 Hz), 131.31 (d, *J* = 2.0 Hz), 130.21, 128.78, 127.0 3 (d, *J* = 4.6 Hz), 125.12 (d, *J* = 3.5 Hz), 123.66, 121.77, 119.72, 117.47 (d, *J* = 38.8 Hz), 116.31, 116.09, 56.77, 56.46, 45.60. HREI-MS: M^+ calcd for $C_{27}H_{23}FN_2O_3$ 442.1693, found 442.1680.

BAP 100: Light yellow powder; Yield: 83%; m.p: 154-156℃; IR (cm^{-1}): 3075(m), 2800(m), 1673(s), 1615(s), 1582(s), 1511(s), 1482(s), 1451(s), 1374(s), 1270(s), 1220(s), 1178(s), 1100(s), 1082(s), 989(s), 920(m), 756(s). ^1H NMR (400 MHz, DMSO) δ 13.46 (s, 1H, -OH), 9.78 (s, 1H, -OH), 8.83 (s, 1H, -N =CH), 8.53 (s, 1H, -OH), 7.68 (s, 1H, -C=CH), 7.67 (s, 1H, -C=CH), 7.57 – 7.45 (m, 4H, $-C_6H_4$), 7.43 – 7.27 (m, 4H, $-C_6H_4$), 6.97 (d, *J* = 8.5 Hz, 1H, $-C_6H_2$), 6.44 (d, *J* = 8.5 Hz, 1H, $-C_6H_2$), 3.80 (s, 2H, $-CH_2$), 3.66 (s, 2H, $-CH_2$), 2.37 (s, 3H, $-NCH_3$). ^{13}C NMR (100 MHz, DMSO) δ 186.49, 164.41, 163.5 2, 160.83 (d, *J* = 249.1 Hz), 151.71, 150.96, 148.76, 136.21, 135.76, 135.42, 1 34.33, 132.81, 131.32 (d, *J* = 1.8 Hz), 130.25, 128.29, 127.02 (d, *J* = 4.9 Hz), 125.12 (d, *J* = 3.4 Hz), 124.69, 123.50, 122.69 (d, *J* = 13.0 Hz), 122.47, 11 6.21 (d, *J* = 21.7 Hz), 112.75, 108.78, 108.30, 56.78, 56.46, 45.60.HREI-MS:

M^+ calcd for $C_{27}H_{23}FN_2O_4$ 458.1642, found 458.1648.

BAP 101: Light yellow powder; Yield: 88%; m.p: 148-150℃; IR (cm^{-1}): 3 055(m), 2939(m), 2845(m), 1672(s), 1619(s), 1599(s), 1562(s), 1486(m), 1452(s), 1293(s), 1271(s), 1177(s), 1134(m), 1030(m), 925(s), 835(m), 755(s). ^{1}H NMR (400 MHz, DMSO)δ 13.49 (s, 1H, -OH), 8.91 (s, 1H, -N=CH), 7.68 (s, 1H, -C=CH), 7.67 (s, 1H, -C=CH), 7.59 – 7.36 (m, 7H, $-C_6H_4$), 7.36 – 7.26 (m, 2 H, $-C_6H_4$), 6.58 (d, J = 8.0 Hz, 1H, $-C_6H_3$), 6.51 (s, 1H, $-C_6H_3$), 3.81 (s, 3H, $-OCH_3$), 3.78 (s, 2H, $-CH_2$), 3.65 (s, 2H, $-CH_2$), 2.37 (s, 3H, $-NCH_3$). ^{13}C N MR (100 MHz, DMSO) δ 186.48, 164.22, 163.85, 163.47, 160.83 (d, J = 249. 0 Hz), 148.84, 136.22, 135.77, 135.34, 134.67, 134.37, 131.95 (d, J = 8.6 Hz), 131.30 (d, J = 2.0 Hz), 130.21, 128.43, 126.98 (d, J = 4.6 Hz), 125.10 (d, J = 3.4 Hz), 123.57, 122.76, 122.64, 116.19 (d, J = 21.7 Hz), 113.40, 107.36, 101.22, 56.79, 56.48, 55.92, 45.61. HREI-MS: M^+ calcd for $C_{28}H_{25}FN_2O_3$ 456.1 849, found 456.1841.

BAP 102: Light yellow powder; Yield: 72%; m.p: 128-130℃; IR (cm^{-1}): 3 083(m), 2935(m), 1674(s), 1619(s), 1589(s), 1466(s), 1274(s), 1254(s), 1181(s), 1100(s), 976(s), 842(s), 759(s). ^{1}H NMR (400 MHz, DMSO) δ 13.03 (s, 1H, -OH), 8.96 (s, 1H, -N=CH), 8.14 (s, 1H, $-C_6H_4$), 7.68 (s, 1H, -C=CH), 7.67 (s, 1H, -C=CH), 7.57 (t, J = 7.7 Hz, 1H, $-C_6H_4$), 7.52 (s, 1H, $-C_6H_4$), 7.47 (s, 4H, $-C_6H_4$), 7.37 – 7.29 (m, 2H, $-C_6H_4$), 7.27 (s, 1H, $-C_6H$), 3.86 (s, 3H, -OC H_3), 3.79 (s, 2H, $-CH_2$), 3.66 (s, 2H, $-CH_2$), 2.38 (s, 3H, $-NCH_3$). ^{13}C NMR (150 MHz, DMSO) δ 186.61, 161.02, 160.87 (d, J = 247.5 Hz), 151.97, 136.7 3, 136.08, 135.87, 135.51, 135.20, 134.36, 132.02 (d, J = 8.7 Hz), 131.66, 131. 35, 130.91, 130.16, 129.85, 129.49, 128.53, 127.04 (d, J = 4.5 Hz), 125.17 (d, J = 3.3 Hz), 123.44, 122.75 (d, J = 12.9 Hz), 122.52, 116.25 (d, J = 21.7 Hz), 57.18, 56.87, 56.52, 45.67. HREI-MS: M^+ calcd for $C_{29}H_{26}BrFN_2O_3$ 548.11 11, found 548.1107.
